# Validation of the CPAP Habit Index-5: A Tool to Understand Adherence to CPAP Treatment in Patients with Obstructive Sleep Apnea

**DOI:** 10.1155/2014/929057

**Published:** 2014-04-27

**Authors:** Anders Broström, Per Nilsen, Benjamin Gardner, Peter Johansson, Martin Ulander, Bengt Fridlund, Kristofer Årestedt

**Affiliations:** ^1^Department of Nursing Science, School of Health Sciences, Jönköping University, 551 11 Jönköping, Sweden; ^2^Department of Clinical Neurophysiology, Linköping University Hospital, 581 85 Linköping, Sweden; ^3^Division of Health Care Analysis, Faculty of Health Sciences, Department of Health and Society, Linköping University, 581 83 Linköping, Sweden; ^4^Health Behaviour Research Centre, Department of Epidemiology and Public Health, University College London, London, WC1E 6BT, UK; ^5^Department of Cardiology, Linköping University Hospital, 581 85 Linköping, Sweden; ^6^Departement of Clinical and Experimental Medicine, Division of Clinical Neurophysiology, Faculty of Health Sciences, Linköping University, 581 83 Linköping, Sweden; ^7^School of Health Sciences, Jönköping University, 551 11 Jönköping, Sweden; ^8^Faculty of Health and Life Sciences, Linnaeus University, 391 82 Kalmar, Sweden; ^9^Department of Medicine and Health Sciences, Linköping University, 581 83 Linköping, Sweden; ^10^Palliative Research Centre, Ersta Sköndal University College and Ersta Hospital, 100 61 Stockholm, Sweden

## Abstract

Long-term adherence to continuous positive airway pressure (CPAP) is low among patients with obstructive sleep apnea (OSA). The potential role of “habit” in sustaining adherence to CPAP use has not been studied. This study aimed to establish the relevance of habit to CPAP adherence, via validation of an adaptation of the Self-Report Habit Index (the CPAP Habit Index-5; CHI-5). Analyses focused on the homogeneity, reliability, and factor structure of the CHI-5 and, in line with theoretical predictions, its utility as a predictor of long-term CPAP adherence in middle-aged patients with OSA. A prospective longitudinal design was used. 117 patients with objectively verified OSA intended for CPAP treatment were recruited. Data was collected via clinical examinations, respiratory recordings, questionnaires, and CPAP devices at baseline, 2 weeks, 6 months, and 12 months. The CHI-5 showed satisfactory homogeneity interitem correlations (0.42–0.93), item-total correlations (0.58–0.91), and reliability (**α** = 0.92). CHI-5 data at 6 months showed a one-factor solution and predicted 63% of variance in total CPAP use hours after 12 months. Based on the satisfactory measurement properties and the high amount of CPAP use variance it explained, the CHI-5 can be seen as a useful tool in clinical practice.

## 1. Introduction

Obstructive sleep apnea (OSA) is a common sleep-related breathing disorder where repeated episodic collapses of the upper airways during sleep cause apneas and/or hypopneas. The resulting sleep fragmentation can cause daytime symptoms, including sleepiness, headaches, and cognitive dysfunction. This condition is termed obstructive sleep apnea syndrome (OSAS) [1]. Apart from the short-term negative consequences due to disturbed breathing, a growing body of evidence indicates that OSAS is also a risk factor for hypertension, cardiac failure, stroke [2, 3], and occupational accidents due to sleepiness. The current treatment of choice is continuous positive airway pressure (CPAP) [4]. Adherence to CPAP treatment is important since sufficient use can eliminate apneas completely and improve sleep quality, excessive daytime sleepiness, and quality of life for both patients and partners. Furthermore, CPAP treatment can reduce morbidity and mortality in cardiovascular diseases, as well as consumption of health care resources [2].

Despite the documented beneficial effects of CPAP treatment, adherence rates tend to be low [5–7]. Initial refusal to engage in CPAP treatment ranges from 8% to 15% and the long-term usage is reported to be between 65% and 80%. In a study by Kribbs et al. [8], less than 50% of patients were considered adherent (i.e., measured as use of CPAP for at least 4 hours per night). Explanations of low and varying degrees of adherence have predominantly focused on technical and physiological aspects [7]. Side effects from treatment, such as dry throat, nasal congestion, and mask leaks tend to be common and are believed to reduce adherence [9–12]. Several technical solutions have been proposed to overcome these problems, such as air humidifiers and different types of devices, yet the increase in adherence has not been proportional to the reduction in side effects offered by these solutions [13]. Objective measures of disease severity are only weakly to moderately associated with CPAP adherence [7].

The difficulties in explaining low CPAP adherence rates have led to calls for more research on the influence of psychological factors on CPAP adherence [6, 9, 14–16]. A few studies have examined psychological factors such as risk perception, attitudes to treatment, outcome expectancies, and self-efficacy [7, 17, 18], which are important constructs in many social-cognitive theories such as the theory of planned behaviour, the theory of reasoned action, and the social cognitive theory [19]. These theories tend to position intention, which summarises various motivational concepts such as attitudes and self-efficacy, as the key determinant of behaviour. However, findings in various fields indicate a rather substantial intention-behaviour gap that implies that behaviour is not consistently guided by motivation [20]. Intentions tend to predict initiation of behaviour but are less predictive of maintenance over time [21]. Within health psychology, “habit” is increasingly attracting interest as a potential mechanism for the maintenance of behaviour [22, 23].

Habitual behaviours are automatic, impulse-driven responses to contextual cues, acquired through repetition of behaviour in the presence of these cues [22]. The context is the environment in which behaviour takes place; the features or cues that trigger action can be anything from physical location, time, and preceding actions to emotions or people [24]. Habit formation offers a useful goal for behaviour change interventions: automatically initiated behaviours may persist over time, even when conscious motivation erodes, which will aid maintenance. As habits form, control over the behaviour is transferred from conscious motivation to environmental cues [25]. Maintenance of the behaviour becomes less reliant on conscious attention and memory processes and instead becomes automatic and “second nature,” proceeding without forethought [25]. Theory predicts that people with stronger habits will be more likely to perform the (habitual) behaviour [26]. CPAP adherence is likely to have the potential to become habitual, given that CPAP is (or should be) used frequently (nightly) in similar settings (the bedroom) [27].

Identifying “habit”—or its absence—in CPAP patients requires a robust and reliable measure of habit strength. The self-report habit index (SRHI) is the most widely applied generic habit strength measure [22, 28, 29]. It has been used with numerous behaviours, including snacking, fruit consumption, engaging in exercise and active sports, watching television, and using a bicycle as a means of transportation [22], but also in adherence to medication [19]. However, no previous study has investigated its usefulness for understanding adherence to CPAP treatment. It would be valuable from a clinical perspective to adapt to the SRHI for a CPAP context, for the purposes of describing the automaticity with which CPAP is used, tracking the formation of CPAP-use habits over time, and predicting long-term CPAP adherence.

Accordingly, the aim of this study was to validate an adapted version of the SRHI called the CPAP Habit Index-5 (CHI-5) in a population of middle-aged patients with OSA. To this end, we sought to establish the internal consistency of the CHI-5, its underlying factor structure, and, in an illustrative application, its utility as a predictor of long-term CPAP adherence. We hypothesised that the CHI-5 would be a homogeneous, reliable, and unidimensional measure predictive of long-term CPAP adherence.

## 2. Materials and Methods

### 2.1. Description of the SRHI

The SRHI is comprised of 12 items concerning three characteristics of habit: 8 items relating to aspects of automaticity (e.g., “Behaviour X is something I do without thinking”), 3 items concerning frequency (e.g., “Behaviour X is something I do frequently”), and 1 item that focuses on the relevance of the habit to self-identity (e.g., “Behaviour X is typically ‘me'”) [29]. A 5- or 7-point Likert-type scale is typically used. Several shorter versions of SRHI have been used with no apparent losses in reliability or predictive validity [30], suggesting that some items may be redundant.

### 2.2. Development of the CPAP Habit Index-5

Three of the authors (AB, PN, and MU) reduced the 12-item SRHI to 5 items for the purposes of achieving a pragmatic tool for potential use in CPAP treatment practice. The selected items were then discussed by a multiprofessional expert panel consisting of three physicians specialized in sleep medicine, three nurses working primarily with CPAP treatment, and a behavioural scientist, as well as two nurse researchers with experience of OSAS. The aim of this discussion was to establish face and content validity of the items. After a consensus decision it was determined to use the 5 items (3 automaticity items and 2 frequency items) in the CHI-5. Previous work has shown that self-identity is not a necessary component of a habit, and so the self-identity item was excluded from our six-item index. A five-point Likert-type scale (1–5) was applied for each item, yielding a possible range for the CHI-5 of 5–25, with a lower score indicating a stronger habit of wearing the CPAP. To strengthen face validity, a group consisting of 20 patients with OSAS answered 5 open-ended questions and was interviewed after performing a pilot test during follow-up visits at the CPAP clinic regarding readability, clarity, and layout with satisfactory results during the development phase.

To secure equivalence when translating the CHI-5 into English all steps in Brislin's model [31] were followed. First two external bilingual adult individuals (i.e., one healthcare professional and one lay person) examined and approved the conceptual structure of the Swedish text. Then two of the authors (AB and PN) translated the scale into English. The English translation was examined by a behavioural scientist with English as a first language (Benjamin Gardner.) and a bilingual group consisting of three sleep experts (i.e., physician, nurse, and technician), as well as two bilingual patients with OSAS, who proposed only a few minor modifications of the wording. One of the authors (Per Nilsen and an external bilingual individual then translated the scale back into Swedish. Finally, the back translation of CHI-5 was carefully examined by the external bilingual group and determined to be equivalent to the original text. The CHI-5 questions are presented in Table 1.

### 2.3. Design and Setting

A prospective longitudinal design was used. One hundred and seventeen eligible patients 18–65 yrs with objectively verified OSA (i.e., based on six channel polygraphy recordings scored according to the American Academy of Sleep Medicines criteria [4]) referred to for CPAP treatment at an Ear Nose and Throat clinic at a county hospital in the southeast of Sweden were recruited. CPAP initiation followed clinical routines and Auto-CPAP, humidifier, and a nasal or full face mask (ResMed; Sweden) were used with the intention to treat during all hours of sleep. There was an initial visit to a physician and thereafter four visits to a nurse during the first year (i.e., initiation meeting and followup after 2 weeks, 6 months, and 12 months.) Exclusion criteria were terminal disease, severe psychiatric disease, dementia, alcohol/drug abuse, or difficulties reading and understanding the Swedish language. The study, following the Helsinki declaration, was approved by the ethics committee (study code M29-07) at the University of Linköping, Sweden, and all participants provided informed consent.

### 2.4. Data Collection

#### 2.4.1. Clinical Variables

At baseline a physician examined the patient and data regarding sleep related symptoms, body composition, blood pressure, medication, and comorbidities were collected. Diagnosis of diabetes mellitus was based on history, current treatment (oral therapy or insulin), or repeated fasting blood glucose values ≥7 mmol/L. Ischemic heart disease was defined as a history of angina pectoris and/or myocardial infarction and/or coronary angioplasty and/or coronary bypass surgery. Respiratory disease was defined as a history of asthma or chronic obstructive pulmonary disease or current treatment with *β* ^2^ agonists and/or inhaled corticosteroids. Objective adherence to CPAP treatment (minutes/night) was obtained from the CPAP device after 12 months. A cutoff of CPAP use >4 h/night was also used to establish adherence.

#### 2.4.2. Self-Rating Scales

The CHI-5 was administered after 6 months. Three other measures were used, for sample description purposes.

The well-validated Epworth Sleepiness Scale (ESS) was used at baseline to measure excessive daytime sleepiness [32]. The eight items (i.e., different daily situations in which the subjects are asked to rate the likeliness of dozing or falling asleep) are rated on a scale of 0–3, where high scores indicate a greater propensity of falling asleep. The total score ranges from 0 to 24 points, with a cut-off of >10 indicating excessive daytime sleepiness.

The side effects to CPAP inventory (SECI) was used after 6 months to measure CPAP side effects [12]. A five-point Likert-type scale, with scores ranging from 1 to 5, is used for frequency, magnitude, and perceived impact on adherence of a list of 15 different side effects. The total score for each SECI scale ranges from 15 to 75 with a higher score indicating a higher frequency, a higher magnitude, and a stronger impact on adherence.

The attitudes to CPAP inventory (ACTI) was used after two weeks to measure attitudes to CPAP treatment [17]. The five items are rated on a scale of 1–5 giving a total score ranging from 5 to 25 with a higher score indicating a more negative attitude to CPAP treatment.

#### 2.4.3. Statistical Processing and Analysis

Normally distributed clinical variables on an interval scale were analysed with parametric statistics. The homogeneity of the items was evaluated by calculating item-total correlations for overlap. Correlations above 0.3 were defined as satisfactory. In addition, Cronbach's alpha if item deleted was evaluated [33]. Ceiling and floor effects were evaluated by inspection of frequency of endorsement for the item response alternatives. The D'Agostino-Pearson test was applied to test the normality distribution of the CHI-5 [34].

Exploratory factor analysis (principal component factoring) was performed on 6-month data to investigate the dimensionality among the items [35]. Data were first examined with Bartlett's test of sphericity (*χ* ^2^(10) = 510.1, *P* < 0.001) and with Kaiser-Meyer Olkin measure (KMO) in each item (0.79–0.96) and all items together (0.86). All these examinations indicated great sampling adequacy. Horn's parallel analysis (1000 repetitions) was conducted to decide number of factors.

Nested linear regression models were conducted to explore if habits could predict long-term CPAP adherence. In an initial model, total hours of CPAP use and days of CPAP use >4 h/night were regressed by habits (CHI-5). To control for theoretically sound predictors of adherence [5], OSA severity (AHI), excessive daytime sleepiness (ESS score), attitudes to CPAP (ACTI), and side effects of CPAP (SECI) were included as covariates in a full model.

All statistical analyses were performed with STATA 13.1 (StataCorp LP, College Station, TX, USA).

## 3. Results

### 3.1. Description of Study Population

Table 2 shows demographic and clinical data of the study population. The mean age of the population was 57.8 (SD 6.7) years and 56% were males. Ischemic heart disease and hypercholesterolemia were the most common comorbidities and occurred among 75% and 34% of the participants, respectively. Mean AHI was 26.7 (SD 19.8). After the six-month follow-up was done 29 patients (i.e., 25% of the initial patients) decided to stop using the CPAP. Before the 12-month follow-up 16 additional patients (i.e., 18% of the remaining patients) decided to drop out. At the 12-month follow-up 20 of the remaining CPAP users did not have data regarding CHI-5 and were therefore not included in the linear regression models. Thus, 52 patients (i.e., 44% of the included patients) remained after 12 months. Table 2 shows CPAP use after 2 weeks, 6 months, and 12 months. The mean CHI-5 score (11.04; SD 5.53) was around the scale midpoint, indicating moderate typical habit strength among the sample.

### 3.2. Is the CHI-5 Internally Consistent?

There was a cumulative, but consistent response pattern for the five CHI items, with the majority of patients scoring strongly agree or agree. No response alternative had a greater frequency of endorsement than 48%. According to the coefficient of variation, the greatest variability was demonstrated for item 3, CV = 0.61 (Table 3). The CHI-5 total score showed a positively skew distributed and deviated significantly from a normal distribution (*χ* ^2^(2) = 9.49, *P* = 0.009). The homogeneity of items was satisfactory with significant (*P* < 0.001) interitem correlations between 0.42 and 0.93 (Table 4) and item-total correlations between 0.58 and 0.91 (Table 3). Internal consistency, measured with Cronbach's alpha, was 0.92 (95% CI = 0.88–0.94) (Table 4).

### 3.3. Does the CHI-5 Have a Unidimensional Factor Structure?

Horn's parallel analysis supported a one-factor solution which was in congruence with the Kaiser criteria with eigenvalues >1. The one factor structure based on data after 6 months explained 76% of the total variance (Table 4). The factor loadings were strong for all items (0.70–0.96) and the uniqueness was in general low and did not exceed the common and critical rule of >0.7 (0.09–0.51).

### 3.4. Does the CHI-5 Predict Long-Term Adherence to CPAP Treatment?

A significant association (*P* < 0.001) was shown between habit strength after 6 months and CPAP adherence after 12 months, for both total CPAP use in hours and days of CPAP use >4 h/night (Table 5). These associations remained when covariates (i.e., OSA severity and excessive daytime sleepiness at baseline, attitudes to CPAP treatment after 2 weeks, and side effects of CPAP treatment after 6 months) were included in the full models. Habit strength after 6 months explained 63% of the variance in total CPAP use in hours after 12 months. The corresponding figure concerning days of CPAP use >4 h/night was 60%. None of the covariates were significantly associated with any of the dependent variables in the full models.

## 4. Discussion

This study aimed to validate an index tapping the habitual nature of CPAP use among CPAP treated patients with OSA. The index, an adapted version of the SRHI called the CHI-5, showed satisfactory validity and reliability, and CHI-5 data at 6 months was strongly predictive of CPAP use at 12 months. Findings suggest that habit is relevant to CPAP adherence and that the CHI-5 is sensitive to habit.

### 4.1. Validity of the CHI-5

The CHI-5 was adapted from the SRHI by a multiprofessional expert panel supported by established CPAP users. Our intention was to develop a parsimonious and conceptually clear tool to capture CPAP adherence habits in clinical practice. Researchers and clinicians have proposed that the effects of habit on behaviour can be attributed to automaticity [19, 30]: while habits arise from and are expressed in repeated performance, the reason that habits prompt behaviour is because they are automated. Commentators have thus reasoned that indicators of frequency are conceptually redundant within a habit index, as the contribution of past behaviour to habit should be adequately reflected by items pertaining to the automaticity with which action proceeds [29]. However, we chose to include behavioural frequency items within the CHI-5 scale, to help distinguish habit from other types of automatic actions which do not develop through repeated performance [30]. Our item selection strategy seems to have been successful, as the five items showed favourable face validity (i.e., comprehensibility, readability, clarity, and layout) among 20 patients with CPAP treated OSAS, and strong item-total and interitem correlations were observed. The composite five-item scale was also found to have a single-factor structure that explained 76% of variation in item scores, indicating unidimensionality. These results suggest that the CHI-5 is a psychometrically sound measurement instrument.

### 4.2. Clinical Applications of the CHI-5

Adherence to CPAP-treatment is a multifaceted problem [11] and low adherence to long-term treatment is well-documented. Theory and empirical evidence suggest that habit strength predicts the frequency with which behaviour is enacted [27], and our data suggested that habit strength predicts long-term adherence to CPAP treatment in patients with OSA. We could explain two-thirds of the variance in total CPAP use in hours after 12 months by using the CHI-5 score after 6 months, though explained variance was slightly lower when using a cut-off for adherence of >4 h/night. Furthermore, the strong impact of habit strength on objective adherence after 12 months was not altered when controlling for objective or self-assessed covariates that have previously been identified as predictors of adherence (i.e., OSA severity at baseline, excessive daytime sleepiness at baseline, improvement of ESS score after 6 months, attitude to CPAP treatment after two weeks, and side effects to CPAP treatment after 6 months).

An increased number of patients with severe OSA will need CPAP over the following years [1]. It is therefore of great importance to identify those patients where habit development might become a problem. Factors that might influence adherence include disease and patient characteristics, treatment titration procedures, technological device factors, and side effects, as well as psychological and social factors [18, 36]. Markers for the severity of OSA, such as the AHI, are of importance for the choice of a suitable treatment but are only weakly correlated with self-reported symptom severity and quality of life [5]. In the present study, although a high degree of dropout was observed, the baseline AHI, daytime sleepiness, and attitude towards treatment did not have any significant association to objective CPAP use after 12 months. As argued by Weaver and Grunstein [5], the patients' perceptions of their life situation may not automatically reveal the objective severity of the illness or the need for treatment and may therefore not be of importance for the development of habits. However, patients describe desires to avoid symptoms, knowledge about risks, fears of negative social consequences, and positive attitudes to CPAP treatment as facilitators for adherence [11]. In the present study attitude to CPAP treatment after 2 weeks did not have a significant association to CPAP use after 12 months. Cognitive interventions (i.e., CBT) have proved to be of importance for adherence [18].

OSA can cause severe impairments of the whole life situation for both the patient and partner before, as well as during the first period of CPAP treatment [5]. Developing a desirable habit (i.e., to start using the CPAP as a routine every night during the first month) or breaking an undesirable habit (i.e., to take up or increase the CPAP use after a period of nonadherence) can be difficult [11] but may be pertinent to promoting adherence. Low habit strength might, according to our findings, have a detrimental impact on long-term CPAP adherence. Verplanken [37] stresses that habit formation takes time and is facilitated by stability and repetition. Habits form in an asymptotic manner, with early repetitions leading to greatest gains in habit, which then reduce over time as a habit strength plateau is reached [38]. The degree of dedication to routine CPAP use during the early stages of formation may influence the sustainability of the behaviour and as long as the patient retains the CPAP device during the early initiation period, habit development will remain possible. The effects of different types of spousal support [39] have been studied in relation to adherence to CPAP and could be of importance for habit formation. We did not measure spousal support, but a supporting spouse might help to create a stable psychosocial environment, as well as positive cues that can be triggered for repetition and habit development. The interaction between healthcare personnel and patient can also be of importance but has not been studied in a habitual perspective. Olsen et al. [40], however, showed that motivational interviewing can improve adherence. Furthermore, communication patterns and shared decision making [41] have proved important for adherence in other patient groups and may be of importance in targeted interventions to develop a habitual CPAP use.

While we sought to validate the CHI-5 partly via exploration of its utility as a predictor of long-term CPAP adherence, the index has other clinically relevant applications. The scale could be administered repeatedly during the initiation process, for example, before each scheduled follow-up visit, to identify which CPAP users who are likely to require extra educational and emotional support. It can together with other instruments, such as SECI and ACTI, add important information regarding the patient perspective especially in the beginning of the initiation procedure (i.e., the early habit formation). CHI-5 is likely to be sensitive to the habit formation process. Although often conceived as a dichotomous variable (habit versus no habit), habit strength is better portrayed on a continuum [42]. A study in which participants were asked to repeat a behaviour daily and to self-report the automaticity of the behaviour found that an abbreviated version of the SRHI was sensitive to increases in habit strength with repetition [38]. The CHI-5 is likely to be useful for offering a potential explanation for low adherence among CPAP patients with low habit strength. Given the strong association observed between self-reported CPAP use habit and CPAP adherence rates, habit formation represents an important goal for CPAP users. While our data illustrate the power of habit to predict behaviour, our design, whereby habit was measured only at 6 months after initiation of CPAP treatment, precluded examination of the processes and duration of CPAP habit formation. Further work is needed to document the processes, as well as factors of importance for habit formation in CPAP.

### 4.3. Limitations

This is, to our knowledge, the first study that examines habits in CPAP treated patients. No other suitable instrument for measuring habits in this context is available. The drop-out rate was fairly high during the study. A larger sample might have led to a greater variation in the response pattern. According to general recommendations for 10 observations per item, the sample size was adequate for the validation analyses of the CHI-5 [34], but the sample was relatively small for regression analyses limiting the possibilities to include other potential predictors (e.g., age, gender, and educational level). Yet, despite this, habit was identified as a significant predictor for long-term CPAP adherence, even where controlling for four covariates, testifying to the unique and strong contribution of habit to CPAP use.

## 5. Conclusion

This is the first study investigating the reliability and predictive validity of a questionnaire measuring habits in relation to CPAP treatment. Acceptable measurement properties indicate that the CHI-5 can be used to measure habits to CPAP treatment in patients with OSA when intervening to improve adherence. Compared to other constructs, habits seem to hold great importance for CPAP adherence. Although more research is needed to show it conclusively, the present study indicates that focusing on habit-strengthening interventions in the education of patients, as well as in the education of healthcare personnel in charge of the CPAP initiation, offers a new and potentially important component for increasing CPAP adherence.

## Figures and Tables

**Table 1:** A description of the CPAP Habit Index-5.

(1) Using the CPAP nightly is part of my routines a normal week	Strongly agree 1	Agree 2	Undecided 3	Disagree 4	Strongly disagree 5
(2) A special reason is needed if I'm not going to use the CPAP during a normal night	Strongly agree 1	Agree 2	Undecided 3	Disagree 4	Strongly disagree 5
(3) I have used the CPAP nightly for a long time	Strongly agree 1	Agree 2	Undecided 3	Disagree 4	Strongly disagree 5
(4) It feels weird not to use the CPAP during a normal night	Strongly agree 1	Agree 2	Undecided 3	Disagree 4	Strongly disagree 5
(5) I use the CPAP more or less automatically during a normal night	Strongly agree 1	Agree 2	Undecided 3	Disagree 4	Strongly disagree 5

**Table 2:** Characteristics of the population before treatment initiation (*n* = 117).

Variables	Total (*n* = 117)
Age, m (sd)	57.8 (6.7)
Gender, *n* (%)	
Male	66 (56)
Female	51 (44)
Education, *n* (%)	
6 years	28 (24)
9 years	28 (24)
15 years	32 (27)
>15 years	24 (21)
Unknown	5 (4)
Marital status, *n* (%)	
Married	92 (79)
Divorced/living alone	18 (15)
Widow/widower	6 (5)
Unknown	1 (1)
Body composition, m (sd)	
Body mass index	30.6 (5.1)
Blood pressure, m (sd)	
Systolic blood pressure	143.4 (18.6)
Diastolic blood pressure	87.8 (11.2)
Comorbidities, *n* (%)	
IHD	88 (75)
Hypercholesterolemia	40 (34)
Diabetes	20 (17)
Respiratory disease	6 (5)
Smoking, *n* (%)	
Smoking	16 (14)
Exsmoker	26 (22)
Nonsmoker	75 (64)
Obstructive sleep apnea, m (sd)	
AHI	26.7 (19.8)
ODI	24.8 (19.2)
Mean saturation	93.3 (1.7)
Self-rated sleep, m (sd)	
Sleep duration, hours	6.8 (1.1)
Daytime sleepiness	
Total ESS score, m (sd)	8.1 (4.4)
ESS ≥ 10, *n* (%)	40 (34)
CPAP use	
Hours/night after 2 weeks (*n* = 117), m (sd)	4.92 (2.3)
>4 hours/night after 2 weeks (*n* = 117), *n* (%)	74 (63)
Hours/night after 6 months (*n* = 117), m (sd)	4.63 (2.9)
>4 hours/night after 6 months (*n* = 117), *n* (%)	87 (74)
Hours/night after 12 months (*n* = 72), m (sd)	5.75 (1.6)
>4 hours/night after 12 months (*n* = 72), *n* (%)	61 (85)

Key: AHI: apnea-hypopnea index; ESS: Epworth sleepiness scale; IHD: ischaemic heart disease; ODI: oxygen desaturation index.

**Table 3:** Item analysis of the CPAP Habit Index-5 after 6 months of CPAP use (*n* = 116)^1^.

Items	Item statistics			Item score distribution				
	Mean (SD)	CV	ITC	1	2	3	4	5
(1) Using the CPAP nightly is part of my routines a normal week	2.02 (1.22)	0.603	0.881	56 (48.3)	25 (21.6)	17 (14.7)	13 (11.2)	5 (4.3)
(2) A special reason is needed if I'm not going to use the CPAP during a normal night	2.16 (1.32)	0.614	0.580	49 (42.2)	34 (29.3)	9 (7.8)	14 (12.1)	10 (8.6)
(3) I have used the CPAP nightly for a long time	2.28 (1.28)	0.561	0.855	46 (39.7)	23 (19.8)	21 (18.1)	21 (18.1)	5 (4.3)
(4) It feels weird not to use the CPAP during a normal night	2.50 (1.33)	0.534	0.716	37 (31.9)	25 (21.6)	23 (19.8)	21 (18.1)	10 (8.6)
(5) I use the CPAP more or less automatically during a normal night	2.09 (1.25)	0.597	0.913	53 (45.7)	25 (21.6)	18 (15.5)	14 (12.1)	6 (5.2)

Total score	11.04 (5.53)	0.501						

CV: coefficient of variation; ITC: item total correlations.

^1^One participant excluded according to missing data in item no 3.

**Table 4:** Homogeneity and factor structure among items of the CPAP Habit Index-5 after 6 months of CPAP use (*n* = 116)^1^.

Items	Interitem correlations					Factor analysis	
	1	2	3	4	5	Factor	Uniqueness
(1) Using the CPAP nightly is part of my routines a normal week	0.818^b^					0.936	0.124
(2) A special reason is needed if I'm not going to use the CPAP during a normal night	0.585***	0.955^b^				0.700	0.511
(3) I have used the CPAP nightly for a long time	0.887***	0.591***	0.918^b^			0.918	0.157
(4) It feels weird not to use the CPAP during a normal night	0.696***	0.421***	0.710***	0.896^b^		0.822	0.324
(5) I use the CPAP more or less automatically during a normal night	0.929***	0.593***	0.846***	0.753***	0.792^b^	0.955	0.088
Explained variance						0.759	
Kaiser-Meyer-Olkin						0.859	
Bartlett test of sphericity						*χ* ^2^ (10) = 510.1, <0.001	
Cronbach's *α*						0.915	
Cronbach's *α* 95% CI^a^						0.878/0.941	

^a^Bias-corrected (based on bootstrapping with 1000 replications)

^b^Measure of sampling adequacy.

^1^One participant excluded according to missing data in item no 3.

***P < 0.001.

**Table 5:** The association between habits after 6 months and CPAP adherence at 12 months (*n* = 52).

Dependent variable	Independent variables	Initial model		Full model	
		*B* (se)	95% CI	*B* (se)	95% CI
Total hours of CPAP use *n* = 52	(i) Habits	−132.3 (14.3)***	−161.1/−103.5	−141.9 (15.6)***	−173.3/−110.5
	(ii) OSA severity at baseline			−4.0 (2.7)	−9.5/1.5
	(iii) Excessive daytime sleepiness at baseline			−11.7 (15.5)	−42.8/19.4
	(iv) Attitudes to CPAP treatment after 2 weeks			5.8 (21.0)	−36.4/48.1
	(v) Side effects of CPAP treatment after 6 months			15.6 (10.6)	−6.0/37.2
	Model statistics	*F*(1, 50) = 85.10, *P* < 0.001, *R* ^2^ = 0.63		*F*(5, 46) = 18.94, *P* < 0.001, *R* ^2^ = 0.67	

Days of CPAP use over 4 h/night *n* = 52	(i) Habits	−18.4 (2.1)***	−22.6/−14.1	−19.9 (2.3)***	−24.5/−15.3
	(ii) OSA severity at baseline			−0.5 (0.4)	−1.3/0.3
	(iii) Excessive daytime sleepiness at baseline			−1.2 (2.3)	−5.8/3.4
	(iv) Attitudes to CPAP treatment after 2 weeks			2.1 (3.1)	−4.1/8.4
	(v) Side effects of CPAP treatment after 6 months			1.6 (1.6)	−1.6/4.8
	Model statistics	*F*(1, 50) = 76.44, *P* < 0.001, *R* ^2^ = 0.60		*F*(5, 46) = 16.60, *P* < 0.001, *R* ^2^ = 0.64	

***P < 0.001.
